# Why Bubbles Coalesce Faster than Droplets: The Effects of Interface Mobility and Surface Charge

**DOI:** 10.1021/acs.langmuir.4c01247

**Published:** 2024-05-15

**Authors:** Ivan U. Vakarelski, Farrukh Kamoliddinov, Sigurdur T. Thoroddsen

**Affiliations:** † Division of Physical Sciences and Engineering, King Abdullah University of Science and Technology (KAUST), Thuwal 23955-6900, Saudi Arabia; ‡ Department of Chemical and Pharmaceutical Engineering, Faculty of Chemistry and Pharmacy, Sofia University,1 James Bourchier Avenue, 1164 Sofia, Bulgaria

## Abstract

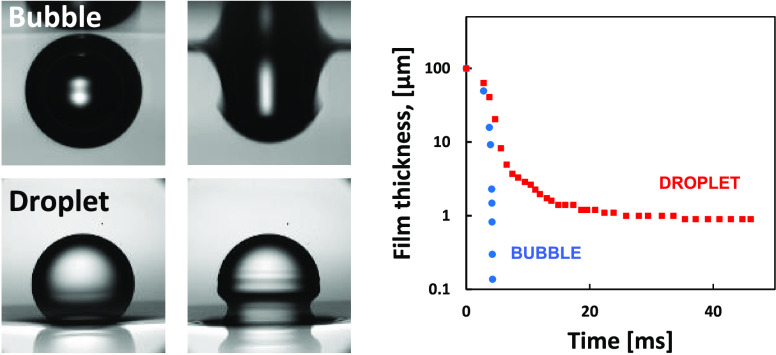

Air bubbles in pure water appear to coalesce much faster compared to oil emulsion droplets at the same water solution conditions. The main factors explaining this difference in coalescence times could be interface mobility and/or pH-dependent surface charge at the water interface. To quantify the relative importance of these effects, we use high-speed imaging to monitor the coalescence of free-rising air bubbles with the water–air interface as well as free-falling fluorocarbon-oil emulsion droplets with a water–oil interface. We measure the coalescence times of such bubbles and droplets over a range of different water pH values (3.0, 5.6, 11.0). In the case of bubbles, a very fast coalescence (milliseconds) is observed for the entire pH range in pure water, consistent with the hydrodynamics of fully mobile interfaces. However, when the water–air interface is immobilized by the deposition of a monolayer of arachidic acid, the coalescence is significantly delayed. Furthermore, the coalescence times increase with increasing pH. In the case of fluorocarbon-oil droplets, the coalescence is always much slower (seconds) and consistent with immobile interface coalescence. The fluorocarbon droplet’s coalescence time is also pH-dependent, with a complete stabilization (no coalescence) observed at pH 11. In the high electrolyte concentration, a 0.6 M NaCl water solution, bubbles, and droplets have similar coalescence times, which could be related to the bubble interface immobilization at the late stage of the coalescence process. Numerical simulations are used to evaluate the time scale of mobile and immobile interface film drainage.

## Introduction

How air bubbles and emulsion droplets coalesce at interfaces determines the properties of many colloidal systems relevant to a wide range of industrial applications and various naturally occurring and biological processes. Examples range from how bubbles aerate the world’s oceans to applications in food, cosmetics, and pharmaceuticals, as well as minerals and crude oil processing. Because of their practical importance and fundamental underlying physics, the interactions involving gas bubbles and emulsion droplets have been extensively investigated using various experimental techniques and theoretical modeling.^1−9^ During the early interaction, the outcome of the collision between two bubbles or droplets depends on the hydrodynamic forces between the approaching bubbles or droplets, while in the later stage, it depends on the surface forces that determine the stability of the thin liquid film formed between the colliding bubbles or droplets.^10−14^ The hydrodynamic force depends on bubble or droplet interface mobility and the surface force on the bubble or droplet surface charge. To investigate the interplay between these fundamental properties, i.e., interface mobility and surface charge, in the present study, we use high-speed camera imaging to monitor the collision of air bubbles with an air–water interface and fluorocarbon-oil droplets with a water–oil interface.

The hydrodynamic interaction between air bubbles or emulsion droplets strongly depends on the tangential mobility of the air–liquid or liquid–liquid interfaces.^15−17^ A clean gas–liquid interface is expected to be fully tangentially mobile and have little resistance to tangential stress. In contrast, the liquid molecules next to a solid interface are immobile, and the fluid velocity is zero, which gives rise to the so-called no-slip boundary condition. However, the presence of even small amounts of a surfactant or other surface-active contaminants can lead to tangential immobilization of the interface due to the Marangoni stress effects.^18,19^ In the case of emulsion droplets, the mobility of the clean droplet interface depends on the viscosity ratio between the droplet and the surrounding liquid. The interface of a droplet of much lower viscosity than that of the surrounding liquid behaves as fully mobile, and that of a droplet of much higher viscosity than that of the surrounding liquid is immobile. Due to smaller velocity gradients and thereby lower viscous stress during the interface approach, bubbles or droplets with mobile interfaces coalesce much faster than immobile interface bubbles or droplets.^16,17^

Determining the mobility of gas bubbles and emulsion droplets in water, the most practically important liquid, has been problematic due to the high affinity of surface-active contaminants or added surfactants to the water interface.^20−23^ Only in recent years have well-controlled experiments been conducted to quantify the surface mobility effect on the collision between bubbles in the water. These experiments include measurements of the bubble’s collision using the dynamic force apparatus technique^19,24^ and high-speed camera tracking of the free-rising bubbles colliding with an interface.^25^ The dynamic force apparatus combines elements of atomic force microscopy (AFM) and a surface force apparatus to allow simultaneous measurement of the force and the film profiles between colliding bubbles. Using this technique, the time scale of the collision between mobile and immobile bubbles in water was quantified.^19^ In a recent study, it was also used to investigate the electrolyte effect on bubble coalescence in water.^24^ Both studies advanced the theoretical modeling of the collision between mobile and immobile bubbles in pure water and electrolyte solutions.

The high-speed camera monitoring of the free-rising bubbles colliding with liquid–air or liquid–liquid interfaces is an alternative technique to quantify the effects of interface mobility. Initial experiments were conducted with bubbles in ultrapure fluorocarbon oils.^26,27^ These experiments demonstrated that in addition to the orders of magnitude faster coalescence of bubbles with a mobile interface than coalescence with an immobile interface, interface mobility could also substantially affect how bubbles and droplets bounce back after the initial collision. Bubbles were found to bounce much more strongly from mobile compared to immobile interfaces.^27^ The lower viscous dissipation explained the stronger bouncing for mobile interfaces when compared to the bouncing from immobile interfaces. Later work also demonstrated this effect for bubbles in pure water bouncing from mobile or immobile water–air interfaces.^25^ The same approach was then used to evaluate the effects of the mobility of bubbles in seawater during the bubble free-rise, bouncing, and coalescence with a seawater–air interface, showing similar effects.^28^

In addition to the hydrodynamic forces, the outcome of the collision between bubbles and droplets at a closer separation distance depends on surface forces, such as the DLVO theory, van der Waals, and the electric double-layer (EDL) force.^29^ The short-ranged van der Waals force is omnipresent and attractive between two similar phases, e.g., between two bubbles or two emulsion droplets, whereas the longer-ranged EDL force depends on the surface charge. Air bubbles and oil emulsion droplets acquire a negative surface charge in pure water, whose strength depends on the pH of the water solution.^30−36^ This surface charge is commonly explained by the spontaneous adsorption of hydroxide ions at the water–air or water–oil interfaces.^30,31^ The surface charge isoelectric point is close to pH 3.0 and increases with the pH, reaching ζ-potentials between −60 and −120 mV at pH 11.0.^30−36^ Following the DLVO theory estimate, such surface charge should prevent emulsion droplets or air bubbles from coalescing (see Appendix A for DLVO force barrier estimates). However, a free-rising bubble in pure water coalesces very fast with a water–air interface,^25^ and emulsions of oil in water are not stable without the addition of surfactant stabilizers. Some prior studies demonstrated that the stability of oil in pure water emulsion can be significantly improved if degassed water is used instead of air-saturated water, implicating the role of hydrophobic forces and cavitation.^37,38^ At the same time, in AFM experiments, the interaction force between small bubbles or oil droplets (*D* ∼ 100 μm) in a low-concentration electrolyte solution in water appears to be entirely repulsive, in agreement with the prediction of the DLVO theory.^5,39^ Generally, slowly colliding bubbles seem to coalesce considerably slower than faster colliding bubbles.^40,41^ It is thus unclear to what extent the spontaneous charging of bubbles or droplets interfaces in pure water can affect the coalescence time.

Herein, we study in parallel and compare the coalescence behavior of bubbles and fluorocarbon-oil droplets. We aim to advance further the understanding of the role of interface mobility and spontaneous surface charging in suppressing or enhancing coalescence. Our prior work on free-rising bubbles colliding with the water–air interfaces has mainly focused on how strongly bubbles bounce from mobile compared to immobile water–air interfaces.^25^ Here, we extend our investigation into the effect of interface mobility on the coalescence times to include various pH pure water. The coalescence time in our experiments is defined as the time the bubble or droplets spend at the interface before the final coalescence. As in our prior investigation, the water–air interface is immobilized by depositing a monolayer of arachidic acid molecules.^25,28^ To test the possible effect of the bubble charge on the coalescence times, experiments are conducted for a range of the water pH: water of low pH 3.0 close to the isoelectric point, atmosphere equilibrated water of pH 5.6, and water of high pH 11.0.

The EDL forces are screened when electrolytes are added to pure water. However, in the case of bubbles, adding a higher concentration of electrolytes delays bubble coalescence.^41−44^ Recent theories speculate that the bubble coalescence inhibition effect is due to the immobilization of the interfaces, which, in turn, is due to the electrolyte concentration gradient related to Marangoni stress effects.^24,45,46^ Here, we further test this hypothesis by measuring the coalescence time of bubbles in a 0.6 M NaCl water solution in the case of both mobile and immobile water–air interfaces. The NaCl concentration of 0.6 M is chosen to be close to that of seawater, e.g., the water in the open seas and oceans.^28^

In the present study, we compare bubble vs fluorocarbon-oil-drop coalescence. There are several reasons to prefer using fluorocarbon-oil droplets over hydrocarbon-oil droplets in the experiments. Fluorocarbon oils are chemically inert and highly resistant to any contamination. Due to their high purity and the high hydrophobicity of the fluorocarbon oil, one can expect similar physicochemical conditions at the pure air–water interface and the perfluorocarbon oil–water interface. The perfluorocarbon liquid we use here is PP1 (perfluoro-2-methylpentane, C_6_F_14_, from F2 Chemicals), which has a density of 1.71 g/cm^3^, which is larger than water density and has a low dynamic viscosity of μ = 0.78 mPa/s^–1^, which is close to that of water. The high density of PP1 secures comparable effective gravity of the free-falling droplets in water to that of the free-rising bubbles in water. The relatively low viscosity of PP1 droplets allows for easier determination of droplet–water interface mobility.

First, we verify the interface mobilities of free-rising bubbles and free-falling PP1 droplets in water by comparing their terminal velocity with theoretical predictions. We then present the coalescence times of bubbles and emulsion droplets with the water interface of various pH. Finally, we use numerical simulations to estimate the time scale for coalescence involving different combinations of mobile and immobile interfaces.

## Experimental and Numerical Methods

### Experimental Setup

**Figure 1:**
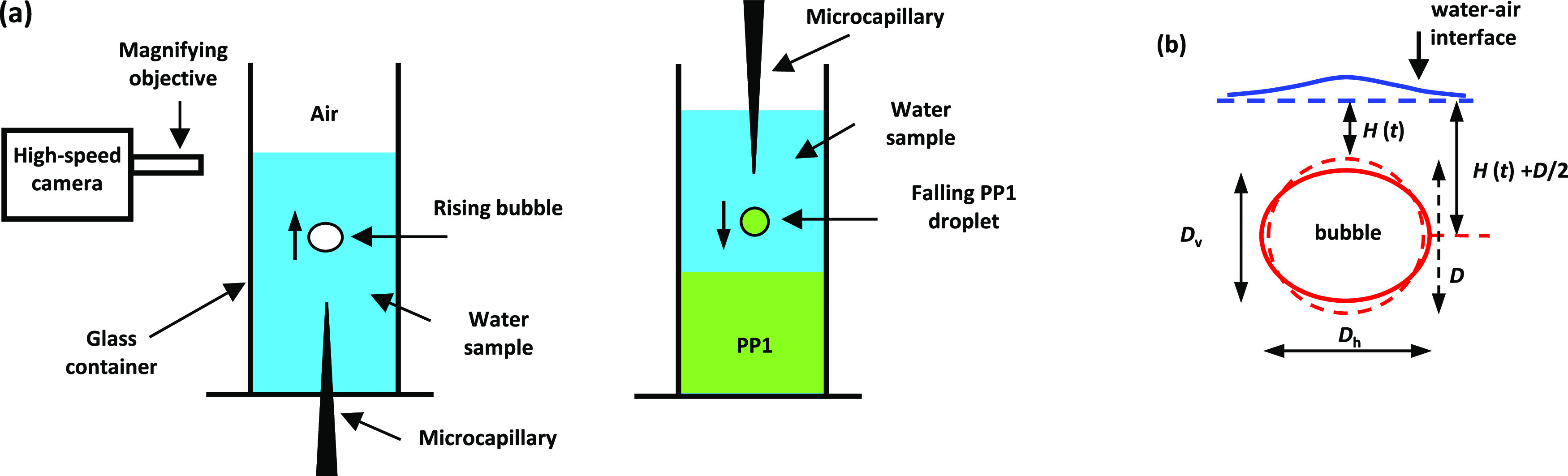
(a) Schematic of the experimental setup for the observation of free-rising bubble collision with the water–air interface (left) or free-falling PP1 droplet onto a water–PP1 interface (right). (b) Schematic of an oblate ellipsoidal bubble of horizontal diameter *D*_h_ and vertical diameter *D*_v_ approaching the water–air interface. The dashed red line indicates the undeformed bubble/droplet of equivalent diameter *D* = (*D*_h_^2^*D*_v_)^1/3^ and the undeformed pool surface by the dashed blue line. The bubble center-of-mass position, relative to the horizontal reference position, is *H*(*t*), indicated with *H* = 0, corresponding to an undeformed bubble in contact with the undeformed interface.

A schematic of the experimental setup used to image a bubble free-rise and coalescence with the water–air interface, or the free-fall of a PP1 emulsion droplet and coalescence with a water–oil interface, is shown in Figure 1a. The setup is adopted from the setups used in our recent related studies.^25−28^ The container was an optical glass cell (Hellma Analytics) with a cross section of 5.0 cm × 4.0 cm and a height of 10.0 cm. A small hole was drilled through the bottom of the cell, into which a glass microcapillary of a 100 μm inner diameter was inserted. The capillary is connected by a plastic tube to a pressure regulator used to generate controlled air-flow pulses. We were able to release bubbles with diameters in the range of 0.6–1.6 mm by using different combinations of air pressure and pulse duration. In the PP1 droplet experiments, the same type of a 100 μm inner diameter microcapillary was mounted above the container and connected by plastic tubing to a 10 mL syringe filled with the PP1 liquid. We produced PP1 droplets in water with diameters ranging from 0.4 to 1.6 mm by applying various pressures to the syringe pistol.

The bubble’s free-rise or droplet’s free-fall and collision with the interface were recorded using a high-speed camera (Photron-SA5) equipped with a long-distance microscope with a 5× magnification objective (Mitutoyo), giving a resolution of 3.3 μm/pixel. The high-speed videos were taken using a typical rate between 1000 and 5000 frames per second (fps) at a shutter speed of up to 1/15,000 s to avoid image smearing and to obtain sharper contrast.

### Bubbles’ and Droplets’ Coalescence Experiments

Bubbles and droplets with an undeformed diameter between 0.4 and 1.6 mm were studied. For this size range, the free-rising bubble or free-falling droplet assumes an oblate ellipsoidal shape, as sketched in Figure 1b. It is convenient to characterize the bubbles or droplets using the equivalent diameter, *D* = (*D*_h_^2^*D*_v_)^1/3^, where *D*_h_ and *D*_v_ are the horizontal and vertical ellipsoidal diameters. In all experiments, the bubbles were released from at least 2.5 cm below the water–air interface to ensure that the bubbles reached terminal velocity before reaching the interface. In the case of droplets, they were released from about 2.0 cm above the water–PP1 interface. The position of the bubble or droplet center-of-mass through time, *H*(*t*), is measured relative to the undeformed water surface (Figure 1b). The time trajectories of the bubble or the droplet center-of-mass positions were determined by image processing of the videos using an in-house developed MATLAB image processing code.

We use Millipore purified water with an internal specific electrical resistance of no less than 18.4 MΩ/cm. NaCl and the arachidic acid (≥99%) were obtained from Sigma-Aldrich. NaCl was baked for 4 h at 500 °C to remove organic contaminants. After equilibrating with the lab atmosphere, the Millipore water acquires a pH of 5.6. The water solution of pH 3.0 was adjusted by adding appropriate amounts of a hydrophilic acid (HCl) solution and the water solution of pH 11.0 by adding a sodium hydroxide (NaOH) solution.

In some experiments, the water–air interface was immobilized by depositing a Langmuir layer of arachidic acid (AAc) on top of the water solution, following a procedure detailed in our recent study.^25^ In short, here, about 8 mL of a 0.1 wt % solution of AAc in chloroform was deposited on top of the water in the glass vessel to get a surface coverage corresponding to an AAc molecular area of about 45 Å^2^. This surface coverage corresponds to a “gas” state of the AAc molecules on the interface and thus does not affect the surface tension while at the same time is high enough to immobilize the interface fully.^25,47^

The perfluorocarbon liquid used was FLUTEC©PP1, a high-performance fluid from F2 Chemicals Ltd., which is mainly composed of perfluoro-2-methylpentane (C_6_F_14_). The PP1 liquid is clear and colorless with density, ρ = 1.71 g/cm^3^ and measured dynamic viscosity, μ = 0.78 mPa/s^–1^ at the laboratory temperature of about 23 °C. Using a Krüss tensiometer, we measured the surface tension of PP1–air of 12.4 ± 0.1 mN/m and interfacial tension of PP1–water of 55.3 ± 0.1 mN/m.

### Gerris Numerical Simulations (GNS)

Following our recent work on bubbles bouncing from interfaces in a perfluorocarbon liquid PP1,^27^ ethanol or water,^25^ and from a water–glass solid surface,^48^ here, in addition to new experiments, we also conduct numerical simulations. We use the freely available open-source code Gerris Flow Solver^49−51^ to simulate both the free-fall of emulsion droplets and the free-rise of bubbles, as well as their collision and coalescence with flat interfaces. This code uses the volume-of-fluid (VOF) method to solve the two-phase incompressible Navier–Stokes equations with free surfaces. Because the code is easy to adapt for an axisymmetric geometry and uses extreme local adaptive grid refinement, the code is very efficient for simulating bubble and droplet dynamics with extremely thin air or liquid films next to the free surfaces during the collision leading to coalescence.

The first type of simulation was conducted to find the terminal free-fall velocity of PP1 droplets in water. The Supporting Figure S1a shows the dimensions of the simulation domain used. This simulation uses the nominal physical parameters of the system: water density is 997.8 kg/m^3^ and water dynamic viscosity is 1.00 mPa/s^–1^. PP1 density is = 1710 kg/m^3^ and dynamic viscosity is 0.78 mPa/s^−1^. The water–PP1 interfacial tension is set at 55.3 mN/m.

**Figure 2:**
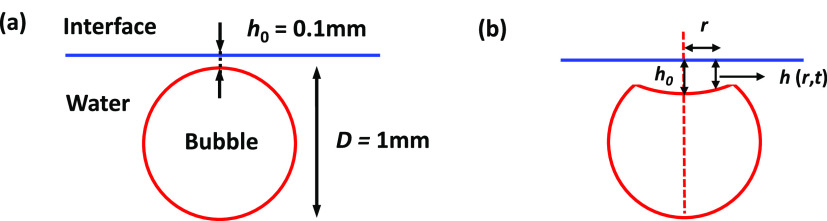
(a) Schematic of the initial position of the bubble and the interface used in the bubble–interface coalescence GNS. (b) Schematic of a bubble coalescing with the flat interface, with *h*(*r*,*t*) tracking the thin liquid film profile (not to scale).

The second type of simulation is conducted to estimate the characteristic time scale of coalescences involving mobile and immobile interfaces. The model system is a *D* = 1.00 mm bubble in water placed under a flat solid wall. As schematized in Figure 2a, the simulation starts with the bubble placed below the top wall and the initial separation between the undeformed bubble top and the wall, *h*_0_ = 100 μm. Here, we are focusing on the final coalescence after the bouncing undeformed bubble is at rest in the beginning of the simulation. In all simulations, the nominal water density is 997.8 kg/m^3^, the viscosity is 1.00 mPa/s^–1^, and the nominal air density is 1.21 kg/m^3^. The water–air surface tension is set to 72.4 mN/m. We use the nominal air viscosity of 1.81 × 10^–2^ mP/s^–1^ to simulate a mobile interface bubble. On the other hand, to simulate a bubble with an immobile interface, we assign the bubble a viscosity ten times that of water, 10.0 mPa/s^–1^, while retaining its low air density.^25,27^ Such an approach for simulating an immobile water–air interface has shown very good agreement with experiments in the case of a bubble bouncing from an immobile water–air interface.^25^ Furthermore, the generic Gerris code allows the application of the no-slip (immobile) and the free-slip (fully mobile) boundary condition at the flat top wall.

To simulate mobile top interface–mobile bubble coalescence, we use free-slip wall and air viscosity bubble; to simulate immobile top interface–mobile bubble coalescence, we use no-slip wall and air viscosity bubble; to simulate mobile top interface–immobile bubble coalescence, we use free-slip wall and high-viscosity bubble; and finally, to simulate immobile top interface–immobile bubble coalescence, we use no-slip wall and high-viscosity bubble.

All simulations start with an adaptive mesh level 11 maximum refinement, i.e., the axisymmetric planar domain is split into squares, where a localized refinement step splits a square into half in both directions. Therefore, the smallest cell size is 2^11^ times smaller than the original domain. As the bubble approaches the wall, the maximum allowed refinement level is gradually increased to better resolve the thin liquid film between the bubble and the interface. The maximum refinement level used here is 17, corresponding to the smallest cell being reduced by 2^17^ to ∼100 nm. Each simulation has been run using 20 cores in parallel within the KAUST IBEX cluster computer nodes (Intel Xeon Gold 6148 Processors). The computational time for the droplets to reach terminal velocity is several hours. The computational time for mobile–mobile interface coalescence is about 2 days, and for immobile interfaces involved coalescence is up to 60 days.

## Results and Discussion

### Bubble and Droplet Interface Mobility

Measuring the free-rise or free-fall terminal velocity of a bubble or an emulsion droplet is a simple and accurate method to evaluate interface mobility. Mobile interface bubbles experience less viscous stress and, therefore, rise faster. The terminal velocity of the bubble depends on the Reynolds number, Re = ρ*DU*/μ, where ρ is the density of the liquid, μ the liquid shear viscosity, *D* is the bubble or droplet diameter, and *U* is the velocity. For the bubble sizes used in the present study, Re ≫ 1, and the rise velocity of the mobile interface bubbles follows the Moore theory, which is valid for a high Reynolds number of deformable bubbles.^52,53^ For the other limiting case of immobile surface spherical bubbles, the rise velocity is given by the empirical Schiller–Naumann relation.^54^ The explicit equations to calculate the terminal rise velocity, using the Moore theory for the mobile case and Schiller–Naumann dependence for the immobile case, can be found elsewhere.^27,53^

**Figure 3:**
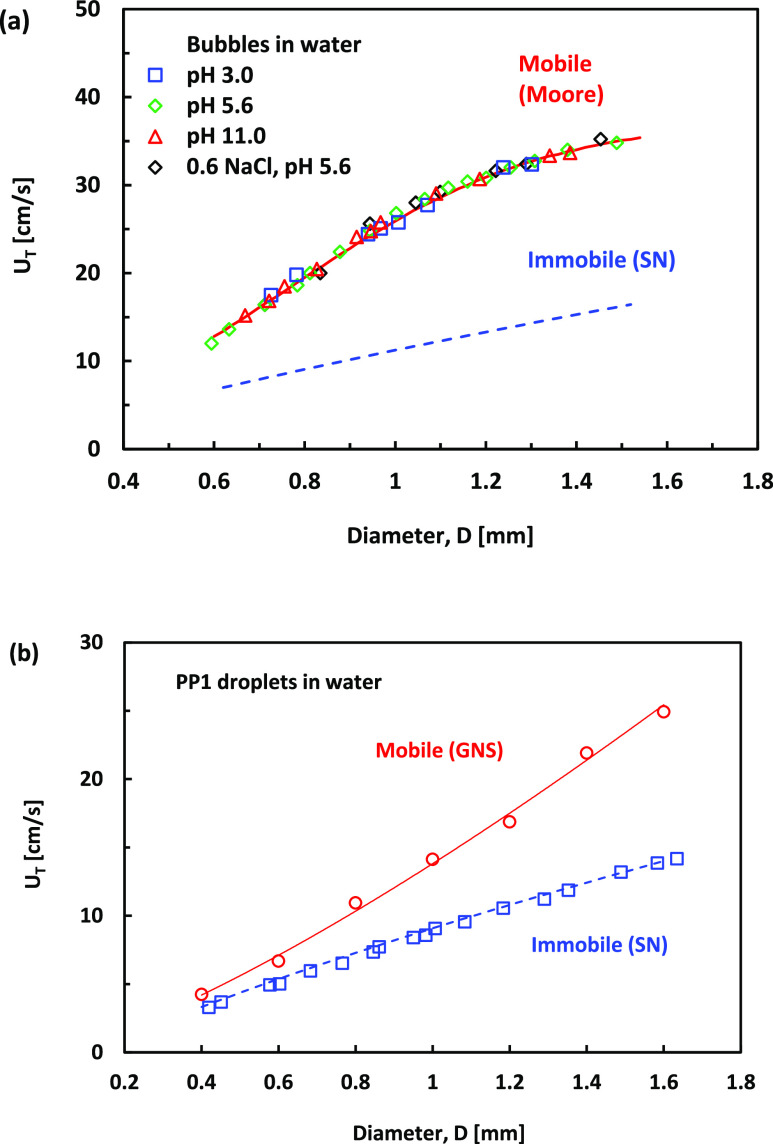
(a) Terminal velocities, *U*_T_, of bubbles free-rising in pure water of pH 3.0 (blue squares), pH 5.6 (green diamonds), pH 11.0 (blue circles), and pH 5.6 with 0.6 M NaCl (black diamonds). The upper red line is the theoretical prediction using Moore’s theory for mobile deformable bubbles, and the lower dashed blue line is the Schiller–Naumann empirical formula for immobile interface spherical bubbles. (b) Terminal velocities, *U*_T_, of PP1 droplets free-falling in pure water (blue squares). The dashed blue line is the Schiller–Naumann empirical formula for immobile interface spherical droplets. The red line is an empirical fit of the GNS (red circle) result for mobile interface droplets.

Although the free-rise velocity of bubbles at small Reynold numbers (Re < 1.00) in water is very sensitive to contamination,^20−23^ the free-rise velocity of bubble sizes 0.6–1.6 mm in pure water is shown to be in good agreement with the Moore theory for fully mobile bubble interfaces.^25,53^ In our recent study of bubble in seawater, we demonstrated that the free-rise velocity of such bubble is not affected by the addition of electrolytes or small amounts of organics at concentrations characteristics for the open seas and oceans.^28^ Here, we further confirm that the free-rise velocity of bubbles in pure water does not change when the pure water pH varies between pH 3.0 and 11.0. This data for the free-rise velocity of 0.6–1.6 mm bubbles at pH 3.0, pH 5.6, pH 11, and pH 5.6 with added 0.6 M NaCl are shown in Figure 3a. This confirms that the interface of the free-rising bubble is always fully mobile for the experimental conditions used in our study.

For emulsions, the viscous stresses at the interface depend on the droplet-to-outer liquid viscosity ratio. For the case of Stokes flow, Re ≪ 1, the transition in the terminal free-rise velocity of a drop from a mobile to immobile interface can be modeled by the Hadamard–Rybczynski velocity:^17^
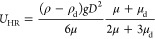
1where *g* is the gravitational acceleration, ρ and μ are the surrounding liquid density and viscosity, respectively, while ρ_d_ and μ_d_ are the droplet values. For droplets of much higher viscosity than the surrounding fluid, this dependence gives the familiar Stokes law for a solid particle, and for droplets of much lower viscosity, the rise velocity is 1.5 higher, as is observed for a clean bubble.

To the authors’ knowledge, for the higher Reynolds number range of the droplets considered in our study, with Re ≫ 1.0, no analytical theory predicts the terminal velocity of droplets with a mobile interface moving in a liquid of comparable viscosity. However, the terminal velocity of such droplets can be modeled using the Gerris numerical simulation (GNS). As in the case of bubbles, the terminal velocity of immobile interface spherical droplets follows the Schiller–Naumann (SN) dependence. Figure 3b compares the measured PP1 droplets’ free-fall velocity with the GNS results for mobile interface droplets and SN relation for immobile interface droplets. Although the GNS predicts up to 2-fold higher terminal velocity for the mobile interface droplets, the experimental data closely follow the immobile interface droplets’ SN relation. Our measurements demonstrate that even for emulsion droplets that are contaminant-free and move in the high Reynolds number range, Re ≫ 1, the interface is much easier to immobilize than bubbles in water of the same purity grade. This difference could be related to the emulsion droplets’ much higher viscosity and density than the air bubbles.

When discussing the interface mobility of a bubble or droplet, one should consider that in addition to the solution conditions, interface mobility depends on the strength of the shear rate along the bubble or droplet interface. As detailed in prior studies, very slowly moving Stokes flow bubbles are immobile even in pure water,^23^ whereas the interface of fast-moving large bubbles or air cavities can be mobile even in the presence of surfactant additives.^17,55^ In this context, bubble mobility determined here holds only for our range of free-rising bubble sizes in the current experiments.

### Bubble Coalescence Time with Mobile and Immobile Water–Air Interface

In recent studies, we demonstrated that millimeter-sized air bubbles free-rising in pure water bounce more strongly from a mobile than an immobile water–air interface.^25,28^ In the following experiments, we investigate the effect of the water pH on the free-rising bubble coalescence times with the interface for both mobile and immobile water–air interfaces in pure water as well as in a 0.6 M NaCl water solution. As detailed in the experimental part, in some of the experiments, the water–air interface is immobilized by depositing a monolayer of archaic acid (AAc).

**Figure 4:**
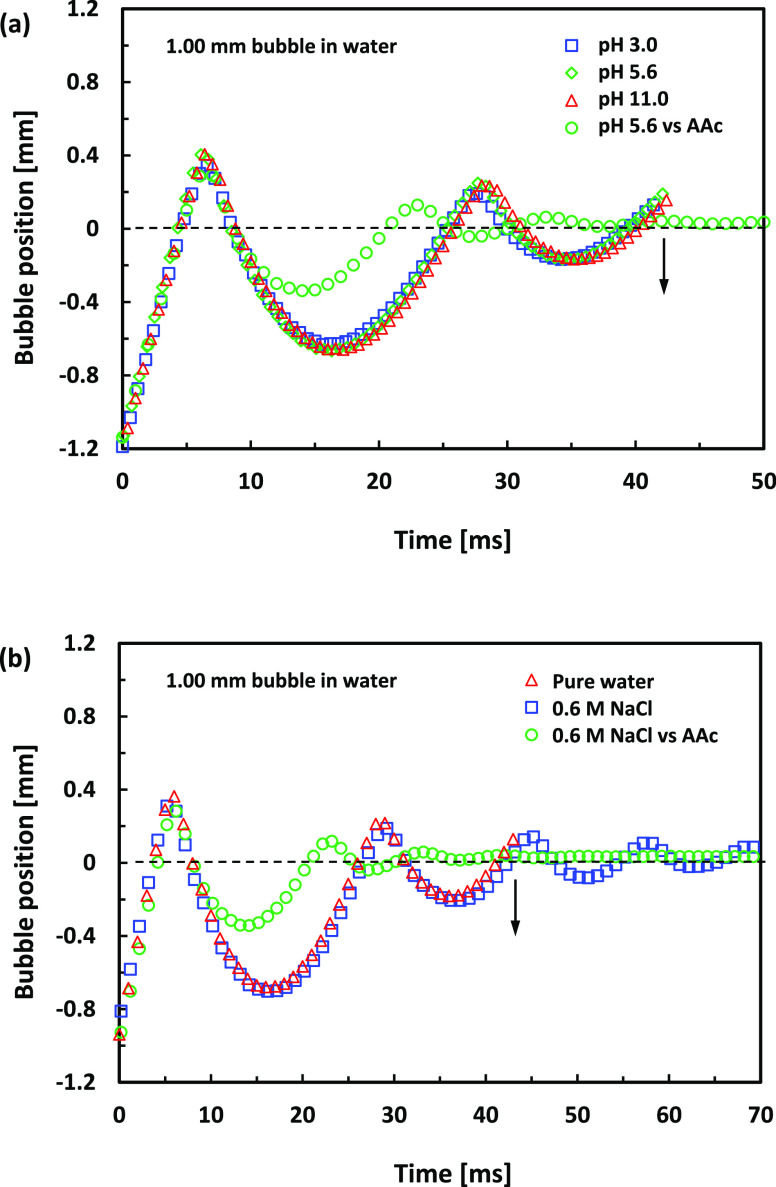
(a) Center-of-mass trajectory of the *D* = 1.00 mm bubble during its bounce from the interface, extracted from Video 1 in the cases of water of pH 3.0 (blue squares), pH 5.6 (green diamonds), and pH 11.0 (blue circles). The trajectory of the same site bubble bouncing from the AAc deposition immobilized water–air interface at pH 5.6 (green circles) is also shown, which is extracted from Video 2. (b) Center-of-mass trajectory of the *D* = 1.00 mm bubble during its bounce from the interface, extracted from Video 3 in the case of pure water of pH 5.6 (red triangles) and a 0.6 M NaCl water solution without (blue squares) or with the AAc deposition (green circles). The arrows in (a) and (b) indicate the approximate time when the bubbles coalesce with the interface.

First, we look at the effect of pH on a pure water–air interface. Video 1 shows an example that shows in parallel the bounce and coalescence of *D* = 1.00 mm bubbles in pure water of pH 3.0, pH 5.6, and pH 11.0. Figure 4a compares the trajectories of the bouncing bubbles extracted from this video. As shown in Video 1 and Figure 4a, there is no significant difference in the bouncing trajectories of the bubbles for this pH range. In all cases, at approximately the same time, the bubbles exhibit a fast coalescence (milliseconds range) with the interface. Such fast coalescence is characteristic of fully mobile interfaces. This result was repeated for the entire investigated bubble size range (0.6–1.6 mm).

These experiments demonstrate that even at pH 11.0, the coalescence of the millimeter-sized bubbles with the free water–air interface occurs without any delay that can be attributed to the spontaneous surface charging of the bubble interface. This is in contrast with the slowly coalescing bubbles in an AFM experiment, in which case the interface charge seems to be high enough to prevent coalescence even in pure water of pH 5.6.^5^ It should be noted, however, that in the case of the AFM bubble experiments, the collision hydrodynamics is consistent with entirely immobile bubble interfaces, whereas in our free-rising bubble collision experiments, the interfaces are fully mobile.^5,25^ One could speculate that the same trace contaminations that immobilize the interface of the slowly coalescing bubble contribute to the surface charge stabilization.

Next, we conduct experiments in which the free-rising bubble collides with the water–air interface immobilized by the deposition of an AAc monolayer. Video 2 compares the bouncing of a *D* = 1.0 mm bubble with a free interface to that of an AAc immobilized interface at pH 3.0, pH 5.6, and pH 11.0. Figure 4a includes the bouncing trajectory with and without the Acc deposit monolayer in water of pH 5.6. As expected, the deposition of the AAc monolayer leads to a lower bounce amplitude of the bubble from the interface.^25^ As in the case of pure water interfaces, there was no measurable difference between the bubble bounce trajectories from the immobilized interface for different pH water (Video 2). However, as shown in Video 2, in all cases of the AAc deposition immobilized water–air interface, the bubble spends some time at the interface before the final coalescence. The time the bubble spends at the interface before the final coalescence is called coalescence time and indicates the drainage rate of the thin liquid film formed between the bubble and the interface.

**Figure 5:**
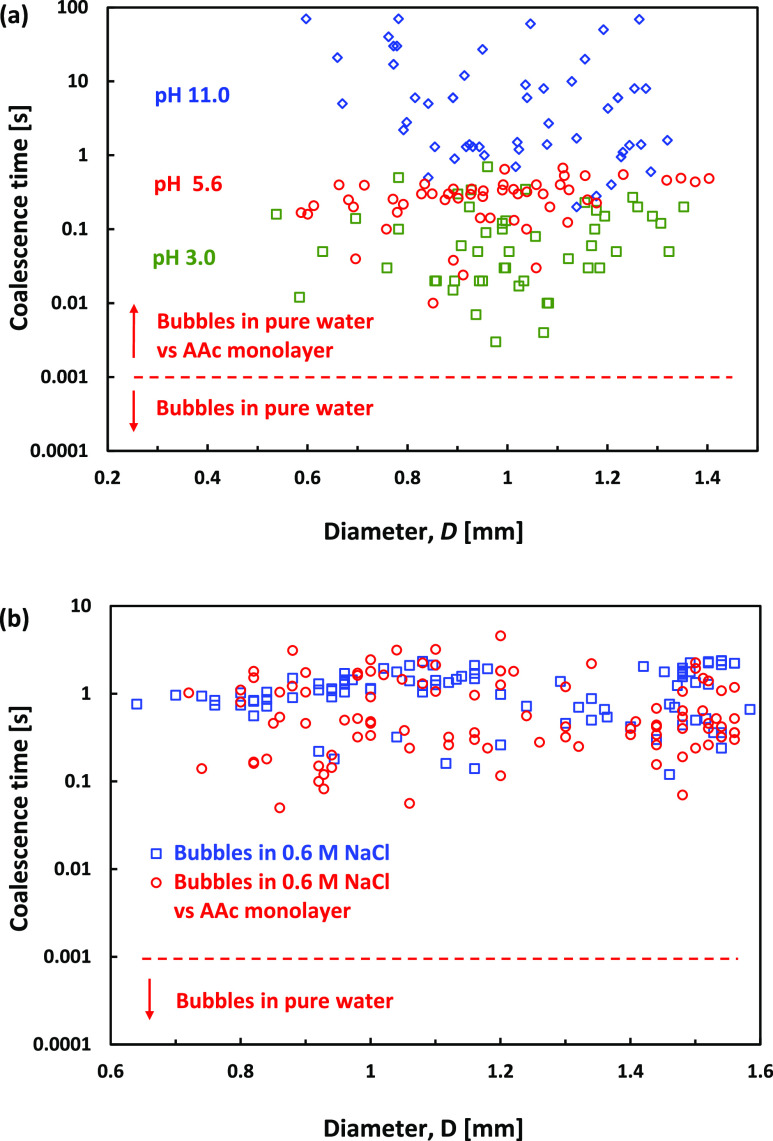
(a) Coalescence times for free-rising bubbles in pure water coalescing with the AAc monolayer deposited on the water–air interface and for the case of water pH 3.0 (green squares), pH 5.6 (red circles), and pH 11.0 (blue diamonds). (b) Coalescence time for free-rising bubbles in a 0.6 M NaCl water solution coalescing with the water–air interface without (blue squares) or with the AAc monolayer (red circles).

Figure 5 shows coalescence time data from multiple free-rising bubbles colliding with the AAc monolayer immobilized water–air interface for pH 3.0, pH 5.6, and pH 11.0. In contrast to the bubble bounce trajectories, which are well reproducible between different runs, there is a significant spread in coalescence times. The relatively large spread in the data is characteristic of such experiments and shows the inherently stochastic nature of the rapture of the thin liquid films.^28,40,41^ Nevertheless, the data show some clear trends. First, in all cases, the coalescence is much longer than in the case of a pure water interface. Second, there is a pronounced dependence of the average coalesce times on the water solution pH. The shortest times are at lower pH 3.0, with an average coalescence time of 0.1 s, followed by pH 5.6, with an average coalescence time of 0.3 s. The longest coalescence time is seen for the highest pH, 11.0, with a much higher average coalescence time of about 12.4 s.

The longer coalescence time of the bubbles colliding with the AAc monolayers can be attributed to two significant factors. The first is the immobilization of the top interface, and the second is the repulsive double-layer force due to the charging of the bubble and the AAc monolayer interface. The extended bubble coalescence time for the higher pH agrees well with the increased surface charge as pH increases. Because the surface charge isoelectric point is expected to be close to pH 3.0,^30−36^ it can be assumed that the coalescence times at that pH 3.0 are characteristic for the case of mobile bubbles coalescing with immobile water–air interfaces while excluding the effect of the interface charge.

In summary, for mobile bubbles coalescing with pure water–air interfaces, there is no time delay due to the presence of the surface charges. On the other hand, when an AAc monolayer is deposited on the air–water interface to immobilize it, then surface charges increase with the increase of the pH, leading to coalescence delay. Although the surface charge on the free-rising bubble does not seem high enough to prevent coalescence with the pure water–air interfaces, its presence is implicated in the coalescence with the AAc monolayer experiments. Why the charge is not preventing the coalescence with a pure water–air interface should be subjected to further investigations.

Finally, we look at the interplay of the water–air interface immobilization in the case of a 0.6 M NaCl water solution. It is well known that high electrolyte concentration inhibits bubbles from coalescing in water.^40−44^ The explanation of this phenomenon and the specific dependence on the electrolyte type has long been debated. Some of the prior hypotheses involve hydration repulsive forces due to ion adsorption,^44^ whereas others attribute the coalescence delay to the bubble interface immobilization due to ion-concentration-gradient-induced Marangoni stress effects.^24,46^

Video 3 parallels the coalescence with the interface of a *D* = 1.00 mm bubble in the case of pure water, a 0.6 M NaCl water solution, and a 0.6 M NaCl water solution with an AAc monolayer deposited on the water–air interface. As shown in our recent seawater investigation, at this NaCl concentration, both the bubble and the water–air interface remain fully mobile during the bubble free-rise and its following bounce from the interface.^28^ This is confirmed by the identical bubble-bouncing trajectories of pure water and 0.6 M NaCl, as shown in Video 3 and Figure 4b. Similarly to pure water, the bubble bounces less from the AAc monolayer with a 0.6 M NaCl water solution. However, as shown in Video 3, in both cases of the NaCl solution without and with the AAc deposition, the coalescence time is much longer compared to that of the pure water case.

Figure 5b compares coalescence times data from multiple bubble experiments in water with 0.6 M NaCl but with and without the AAc monolayer. In both cases, the coalescence times are much longer than in pure water. The average coalescing time for the 0.6 NaCl M solution without AAc deposition is about 1.18 s, and with the AAc monolayer, it is only slightly lower at 0.82 s. The observation that the immobilization of the water–air interfaces in the case of the 0.6 M NaCl solution did not lead to a further increase in the coalescence times is consistent with the hypothesis that the delayed coalescence is due to the immobilization of the interfaces at the final stage of the thin liquid film drainage.^24,28^

### PP1 Droplets Coalescing with the PP1–Water Interface

Next, we investigate the coalescence of free-falling PP1 emulsion droplets onto a water–PP1 interface using the same water solutions as in the bubble experiments. Video 4 shows an example of the PP1 droplet free-falling and coalescing with the water–PP1 interface in the case of pure water of pH 5.6. It is seen that following the initial collision and a few weak bounces, the droplet comes to rest next to the interface, where it sits for some time before the intermediate liquid film ruptures and the final coalescence occurs. As in the case of the bubble experiments, we refer to this time as coalescence time. This should not be confused with the time of the rapid coalescence motions seen in the coalescence cascade at the end of the video.^56,57^ As exemplified in Video 4, the coalescence is much slower than for bubbles in pure water (seconds vs milliseconds).

In addition to the mobility of the interface, another contributing factor for the longer coalescence time of the droplets compared to the bubbles could be the repulsive electric double-layer force (EDL) due to the interface charge combined with the lower attractive van der Waals force for the PP1–water–PP1 system compared to the air–water–air system (see Appendix A). As in the case of bubbles, to determine the effect of the spontaneous surface charge, we conducted experiments for droplet coalescence in water of pH 3.0, pH 5.6, and pH 11.0. An alternative way to suppress the surface charge is by conducting experiments in higher electrolyte concentrations, which was the 0.6 M NaCl water solution in our case.

**Figure 6:**
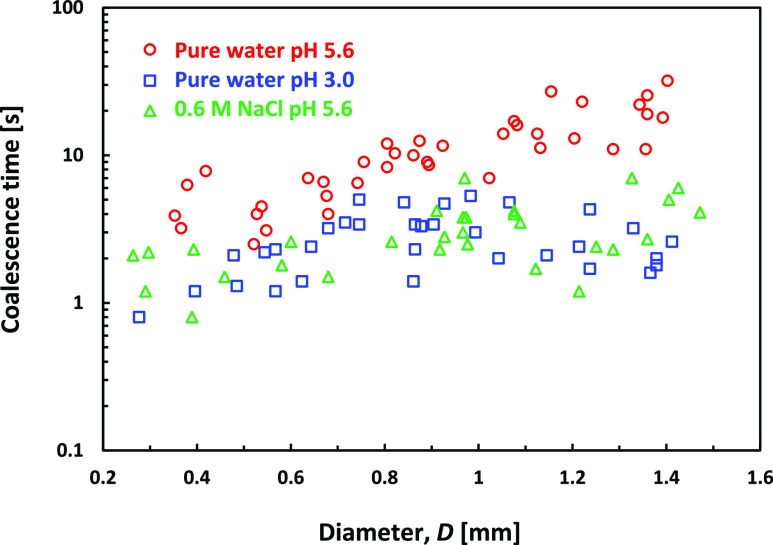
Coalescence times for PP1 droplets with the water–PP1 interface for the case of droplets free-falling in the pure water of pH 5.6 (red circles) and pH 3.0 (blue triangle) or in the 0.6 M NaCl water solution of pH 5.6. For water of pH 11, the droplets do not coalesce and remain sitting on the interface.

Figure 6 summarizes data from multiple experiments for the PP1 droplet coalescence conducted in pure water at pH 3.0, pH 5.6, and 0.6 M NaCl water solutions. Experiments in pH 11.00 resulted in no coalescence and are discussed below. The characteristics coalescence times in pure water of pH 5.6 are in the seconds range, increasing from a few seconds for the smaller droplets of a 0.4 mm diameter to about 10–20 s for the largest 1.4 mm diameter droplets. Figure 5 shows that suppressing the surface charge by either lowering the water pH to 3.0 or adding 0.6 M NaCl results in a similar decrease in the average coalescence time to about 3.0 s. As such, these shorter coalescence times are the typical coalescence times of PP1 droplets in water, for which the repulsive EDL interaction does not delay the film drainage process.

We note that since the droplet–water interface is already immobile, the observation that the addition of 0.6 M NaCl only shortens the droplet’s coalescence times due to the screening of the EDL force provides further support for the hypothesis that the extended coalescence time of bubbles in a 0.6 M NaCl water solution is due to the interface immobilization at the final stage of the film thinning. The characteristic coalescence times for droplets in the 0.6 M NaCl water solution are of the same magnitude as the for bubbles in the 0.6 NaCl water solution (average time of 3.2 s for droplets and of 1.1 s for bubbles), and these are typical times for the coalescence of immobile interfaces. The lower magnitude of the attractive van der Waals force can explain the relatively longer coalescence time for the droplets compared to bubbles in these conditions (Hamaker constant, *A* = 5.6 × 10^–20^ J for the water–air–water, whereas for PP1–water–PP1, *A* = 0.3 × 10^–20^ J, Appendix A).

**Figure 7:**
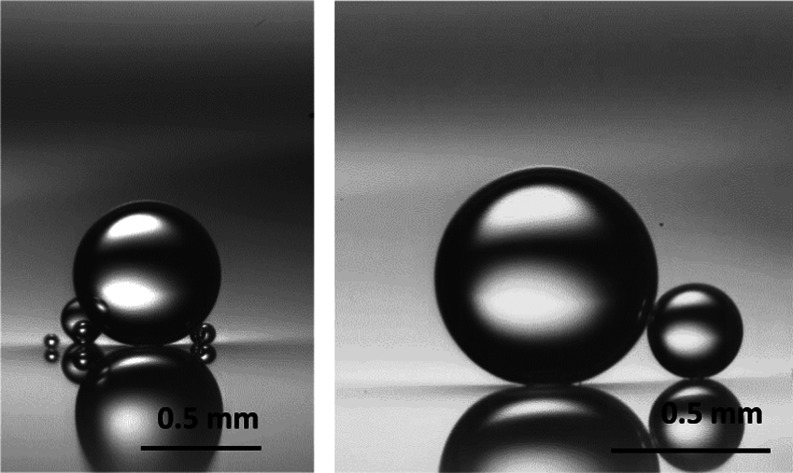
Video snapshots of PP1 emulsion droplets in water of pH 11 sitting at PP1–water interfaces taken at about 2 h after the droplets settled on the interface.

Similar to the experiment of bubble coalescence with the AAc deposited monolayer in the water of pH 11.0, the experiment with PP1 droplets in the water of pH 11.0 showed a much-extended coalescence time compared to the lower pH water solutions coalescence. In fact, in the water of pH 11.0, following the collision of the free-falling droplets with the interface, the droplets were found to stay at the interface without coalescing. Figure 7 shows a snapshot of PP1 droplets of various sizes staying in the PP1–water interface without coalescing several hours after deposition. It is seen that the droplets did not coalesce with the interface or between adjacent droplets. Apparently, in the case of fluorocarbon droplets, the surface charge at pH 11.0 provides a long-time stabilization through the EDL repulsive force. Although prior studies have shown that a surfactant-free oil droplet emulsion can be formed using degassed water,^37,38^ the present result suggests that in the case of fluorocarbon droplets, the stabilization can be achieved by simply using a high-pH water solution. The long-term stability of high-pH-stabilized oil droplet emulsions will be subjected to future investigations.

### Numerical Simulations of Mobile and Immobile Interfaces Coalescence

In recent studies, we have demonstrated the efficiency of the GNS in simulating free-rising air bubbles and emulsion droplets and the bouncing trajectories from solid and deformable interfaces.^25,27^ GNS is far more computationally demanding compared to analytical models such as the force balance model, which was used to simulate a bubble bouncing from an interface.^53,58^ At the same time, GNS has the advantage of explicitly capturing for hydrodynamics of the surrounding flow and was shown to accurately predict not only the bouncing trajectory but also the complex bubble shape evaluation during the bounce.^25,48^ Most recently, by comparing such simulations with interferometric data, we demonstrated that GNS can accurately predict the shape of the thin liquid film formed between a bubble and the solid surface during the bouncing of a free-rising bubble in water from a flat glass plate.^48^

Here, we use GNS to estimate the time scale of the coalescence between an air bubble or emulsion droplets and a flat interface in the case of both mobile and immobile interfaces. As detailed in the Experimental and Numerical Method section, our model system is a *D* = 1.00 mm air bubble in water placed under a flat wall with initial bubble–wall separation, *h*_0_ = 0.10 mm (Figure 2a). We consider four cases of bubble collision with the interface: mobile bubble with a mobile interface, mobile bubble with an immobile interface, immobile bubble with a mobile bubble, and finally, immobile bubble with an immobile interface.

**Figure 8:**
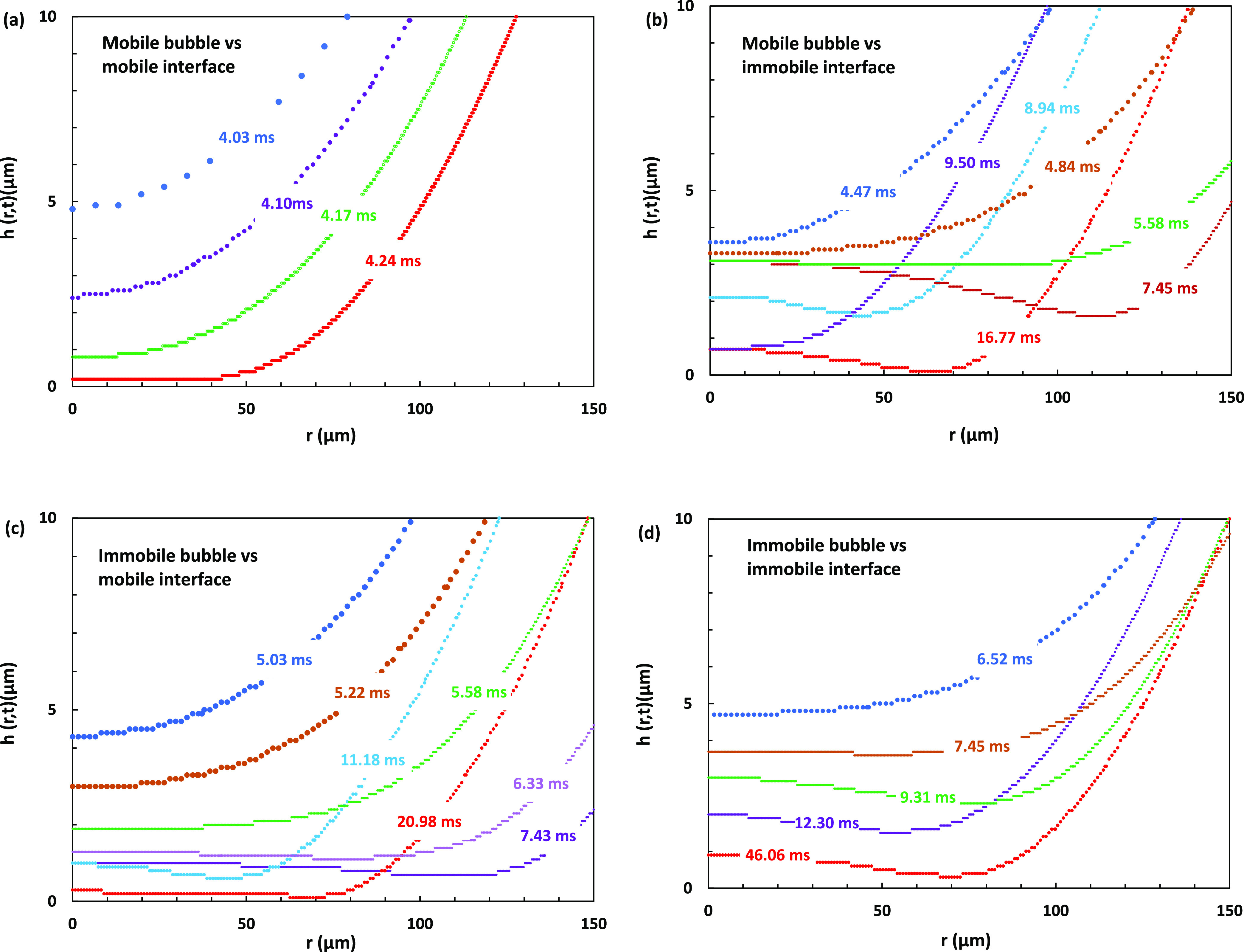
Numerically simulated film profiles *h*(*r*,*t*) for a *D* = 1.00 mm bubble in water approaching a flat interface under different mobility conditions: (a) mobile bubble vs mobile interface, (b) mobile bubble vs immobile interface, (c) immobile bubble vs mobile interface, and (d) immobile bubble vs immobile interface. Approach times in ms are indicated for each profile. The initial position of the undeformed bubble is 0.1 mm below the interface (Figure 2a). The data point density reflects the simulation refinement mesh level used, with higher density corresponding to a higher refinement level. Only the right sides of the symmetric profiles are shown.

**Figure 9:**
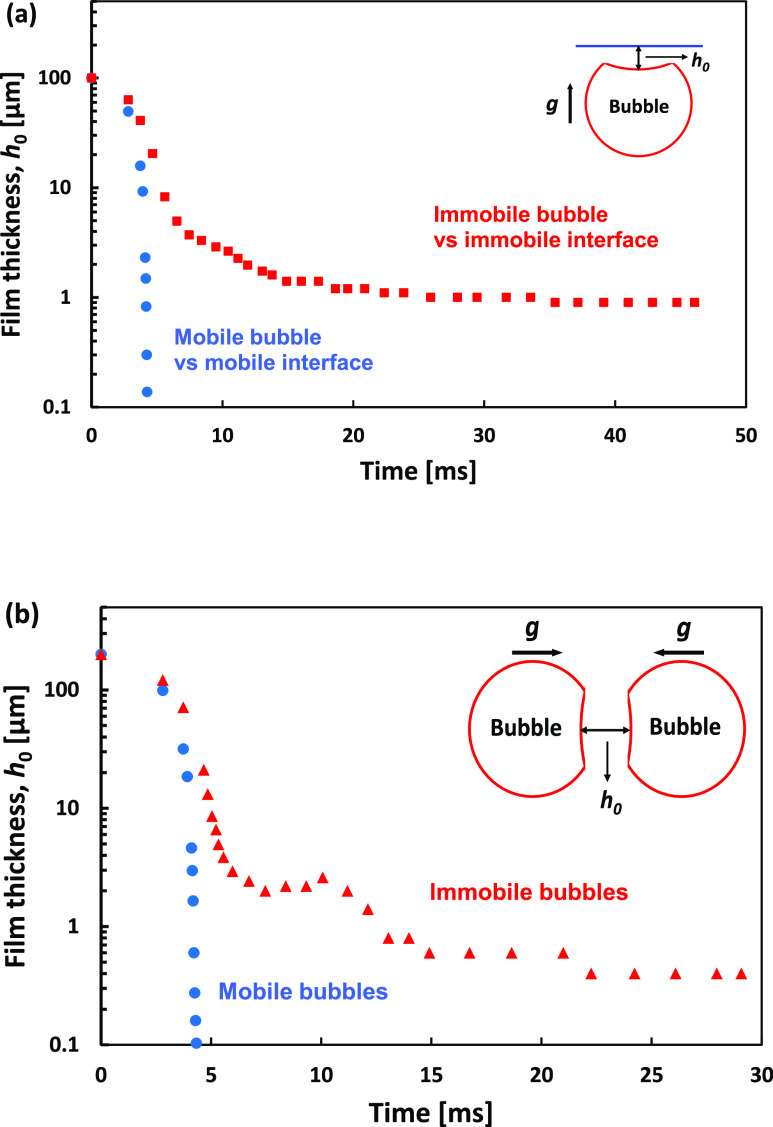
(a) Thinning of the liquid film thickness *h*_0_(*t*) at the axis of symmetry above the bubble vs time, taken from the Gerris simulations. Comparison between the cases where the bubble and the interface are both mobile (blue circles) vs both immobile (red square). (b) Comparison of thinning of the liquid film thickness, *h*_0_(*t*) at the axis of symmetry between two mobile (blue circles) vs two immobile (red triangles) bubbles accelerated toward each other with the gravity acceleration, *g*. Due to the symmetry, the separation between the mobile bubble equals two times the separation in the mobile bubble vs mobile surface case and between the immobile bubble equals two times the separation in the immobile bubble vs mobile surface case.

Results for the four case simulations are presented in Figure 8 as the profiles of the thin liquid film above the bubble approaching the interface, *h*(*t*,*r*), as defined in Figure 2b. Figure 9a contrasts the progression with time of the thickness of the thin liquid film, at the axis of symmetry *h*_0_(*t*), for the mobile bubble against the mobile interface case with the immobile bubble against an immobile interface. In Figure 9b presentation, we take advantage of the fact that due to symmetry, the simulation of mobile bubble coalescence with the mobile interface and immobile bubble coalescence with the mobile interface is also identical to the simulation of two mobile or two immobile 1 mm bubbles’ coalescence when initially separated by *h*_0_ = 0.2 mm and accelerated toward each other with the gravity acceleration (Figure 9b inserts).^27^

The most important feature seen in the simulations in Figures 8 and 9 presentations is the order of magnitude reduction in the thinning rate of the liquid film when both interfaces are mobile, compared to the cases involving immobile interfaces. During the initial approach of the bubbles to the interface, up to *h*_0_ ≈ 5 μm, there is not much difference in the approach rate between the four cases. However, following this, the case of a mobile bubble approaching a mobile interface takes only 0.2 ms for the thin liquid film to collapse from *h*_0_ ≈ 5 μm to *h*_0_ ≈ 0.1 μm (Figure 8a). In contrast, for the immobile bubble approaching an immobile interface, it takes more than 40 ms for the film to thin from *h*_0_ ≈ 5 μm down to *h*_0_ ≈ 1 μm (Figure 8d). The drastic difference in the film thinning rates is also demonstrated in Figure 9a for the bubble vs interface case and Figure 9b for the two colliding bubble case. Another important observation is that the thinning of the film for the mobile interfaces happens with little flattening of the front of the bubble and without the characteristic dimple in the thin liquid film (Figure 8a), which is observed for the other cases (Figure 8b–d). The final film rupture of the films is expected for film thicknesses below 0.1 μm and will depend on the surface forces, such as van der Waals and EDL, which are not included in the Gerris simulation.

Because of the slow progression of the film thinning in cases involving immobile interfaces, these simulations require long computational times (up to two months). Alternatively, the last stage of the film thinning and the final rupture could be resolved more efficiently using analytical models such as the Stokes–Reynolds–Young–Laplace (SRYL) model,^5,6^ which also has the advantage of factoring in the surface forces disjoining pressure. However, our simulations could be used to confirm the analytical models, particularly the coalescence case for mobile against mobile interfaces, to which the SRYL model has only recently been extended.^19^

## Conclusions

Here, we have investigated the coalescence times for free-rising air bubbles when they approach mobile or immobile water–air interfaces, as well as free-falling PP1 fluorocarbon-oil emulsion droplets approaching water–oil interfaces in pure water of pH 3.0, 5.6, and 11.0 and a 0.6 M NaCl water solution. Our experiments indicated that the lead factor for the order of magnitude difference in the coalescence rates between air bubbles and emulsion droplets in pure waters is the difference in mobility of the water–air and water–oil interfaces, respectively. The coalescence of air bubbles with a clean water–air interface was very fast (milliseconds) and representative of coalescence at a mobile interface. The coalescence of PP1 droplets with a water–oil interface was much slower (seconds) and represented immobile–immobile interface coalescence.

A secondary factor for the bubble and oil droplet coalescence rates in pure water was the solution pH-related water–air and water–oil interfaces’ spontaneous surface charge. Although the bubble coalescence with a clean water–air interface shows no evidence of the coalescence delay, due to the surface’s charging, bubbles coalescing with the immobile water–air interface significantly increased the coalescence time as pH increased. The coalescence times’ dependence on the surface charge was also observed for the PP1 oil droplets; in this case, we found that for pure water of pH 11.0, the spontaneous surface charge could completely prevent PP1 droplets from coalescing with the interface.

In the case of coalescence in a 0.6 M NaCl water solution, the observation that the coalescence times of bubbles against mobile interface are similar to that against immobile interfaces and of the same magnitude as the coalescence time of PP1 droplets in 0.6 M NaCl water solutions is supportive of the hypotheses that the delayed bubble coalescence in a high electrolyte solution is due to air–water interface immobilization at the later stage of the thin liquid film thinning.^24,28^

Model GNS of bubble coalescence with interfaces clearly demonstrated the orders of magnitude faster coalescence rates when both interfaces are mobile compared to immobile interfaces. These simulation results can be further used to verify and develop more efficient simulation approaches and analytical models.

## Figures and Tables

**Figure 10:**
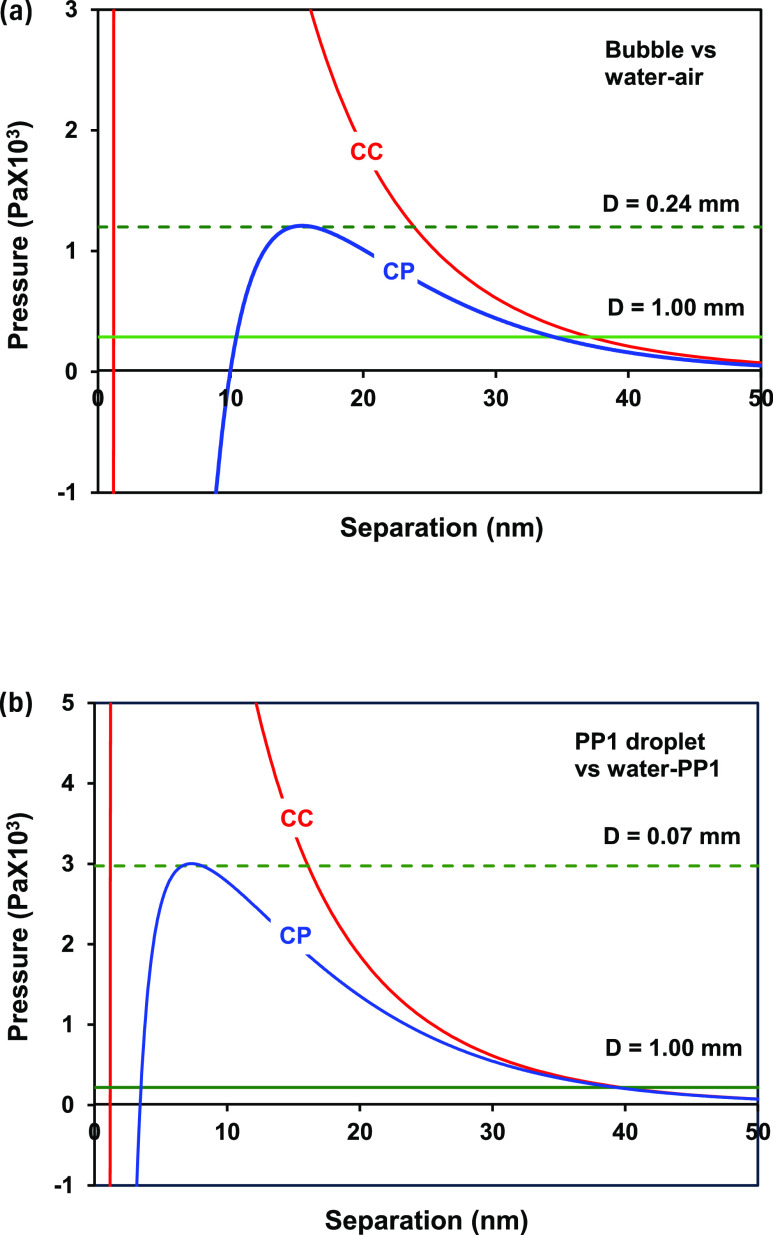
DLVO disjoining pressure between flat interfaces constructed from the sum of the van der Waals interaction and an EDL force in the limiting case of constant surface potential (CP) and constant surface charge (CC) at an electrolyte strength of 10^–3^ M and surface potential, φ_0_ = −30 mV. (a) For the air–water–air system, we use Hamaker constant *A* = 5.6 × 10^–20^ J. (b) For the PP1–water–PP1 system, *A* = 0.3 × 10^–20^ J. The horizontal solid and dashed lines show the Laplace pressure in the system for the indicated bubble (a) or droplet (b) diameters.

## Appendix A: DLVO Force Barrier

Here, we estimate the spontaneous surface charge’s ability to prevent coalescence between a deformable bubble or droplet and an interface using the framework of the classical DLVO theory.^29^ The theory of interaction between deformable bubbles and droplets is well established.^6^ The DLVO disjoining pressure between two flat interfaces is constructed as the sum of the attractive van der Waals force and the repulsive EDL force. To obtain an estimate for the lower and higher limit case repulsive barrier maximum, the EDL force was calculated for constant surface potential (lower limit) and constant surface charge (higher limit) boundary conditions. Calculations were done by numerically solving the Poisson–Boltzmann equation using the algorithm of Chan et al.^59^

If the interaction between the deformable interfaces is fully repulsive, the surface separation reaches a limiting value when a bubble or droplet approaches each other or an interface. This is because the interface starts to flatten as the repulsive force disjoining pressure approaches the Laplace pressure across the bubbles or droplets, Π(*D*_*L*_) = 4γ/*D*, where *D*_L_ is the limiting separation of two flat air–water or oil–water interfaces and γ is the interfacial tension.

Figure 10a shows an example of how the DLVO disjoining pressure changes with the separation distance for a flat air–water interface and Figure 10b shows it for a flat PP1–water interface. The surface potential is taken to be φ_0_ = −30 mV, which is at the lower limit for the experimentally measured ζ-potentials for droplets or bubbles at pH 5.6–11.0.^30−36^ Calculations are done for a 10^–3^ M 1:1 electrolyte, which is the expected electrolyte concentration in pure water at pH 11.0. In calculating the attractive van der Waals force, for the case of the air–water–air system, we use the literature value for the Hamaker constant, *A* = 5.6 × 10^–20^ J, and for the PP1–water–PP1 system, *A* = 0.3 × 10^–20^ J.^29^ In each graph, the straight horizontal solid and dashed lines indicate the Laplace disjoining pressure for different-sized bubbles or droplets. In both cases, the DLVO force barrier readily exceeds the Laplace pressure for the bubble or droplet sizes used in our study. Thus, using DLVO intuition, one should expect that the spontaneous surface charge of the bubbles or droplets, at pH 5.6 or higher, should prevent coalescence with the interfaces for the bubbles and droplet sizes used in our investigation.

## Supporting Information Available

Computational domain (Figure S1) (PDF)
Video comparing *D* = 1.00 mm bubble bouncing and coalescence with the pure water–air interface in the case of water pH 3.0 (left), pH 5.6 (middle), and pH 11.0 (right); the videos are shot at 5000 fps and played at 30 fps (Video 1) (MP4)
Video comparing *D* = 1.00 mm bubble bouncing and coalescence with the water–air interface in the case of (from left to right) the pure water–air interface, water pH 5.6, or water with the AAc monolayer in the case of water with pH 3.0, pH 5.6, and pH 11.0; the videos are shot at 1000 fps and played at 30 fps (Video 2) (MP4)
Video comparing *D* = 1.00 mm bubble bouncing and coalescence with the water–air interface in the case of pure water of pH 5.6 (left), a 0.6 M NaCl water solution of pH 5.6 without the AAc monolayer (middle), or with the AAc monolayer (right); the videos are shot at 1000 fps and played at 30 fps (Video 3) (MP4)
Free-fall and coalescence with the water–PP1 interface of a *D* = 1.00 mm PP1 emulsion droplet in pure water of pH 5.6; the video is shot at 1000 fps and played at 30 fps (Video 4) (MP4)
