# Supplementary material for: Why Bubbles Coalesce Faster than Droplets: The Effects of Interface Mobility and Surface Charge
Source: Langmuir. 2024 May 15;40(21):11340–51. doi: 10.1021/acs.langmuir.4c01247 (PMC11140758; doi:10.1021/acs.langmuir.4c01247)
Supplement: Supplementary file 1 — la4c01247_si_001.pdf [file la4c01247_si_001.pdf]

SUPPORTING INFORMATION

Why bubbles coalescence faster than droplets: the effects of the interface mobility, and the surface charge

Ivan U. Vakarelski[1,2], Farrukh Kamoliddinov[1] & Sigurdur T. Thoroddsen[1]*

[1] Division of Physical Sciences and Engineering,

King Abdullah University of Science and Technology (KAUST), Thuwal 23955-6900, Saudi Arabia.

[2]Department of Chemical and Pharmaceutical Engineering, Faculty of Chemistry and Pharmacy, Sofia University,1 James Bourchier Avenue, 1164 Sofia, Bulgaria.

Table of content:

Supporting Figure S1. Computational domain 2

Number of pages: #2

Number of figures: #1

* CORRESPONDING AUTHOR:

Ivan U. Vakarelski, E-mail: ivakarelski@gmail.com

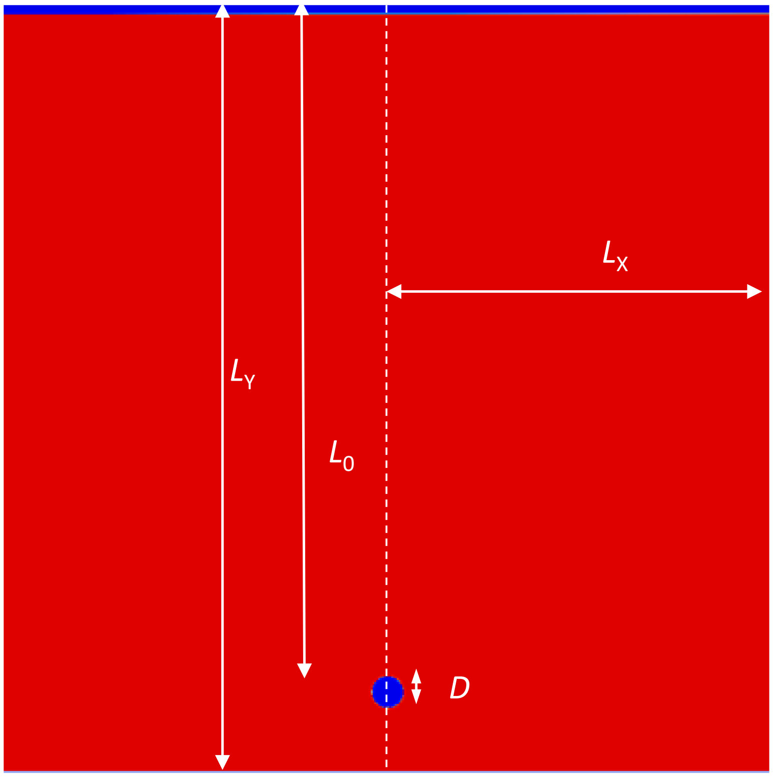

Supporting Figure S1. Computational domain with initial droplet position in the GNS of a PP1 droplet free fall in water. Red is water liquid and blue circle is PP1 droplet, blue line at the top is the wall. D = 1, L_x = 13.5, L_Y = 27, L_0 = 21. In the case of bubble collision with the wall L_0 = 0.1. In the case of free-falling droplet gravity acceleration is oriented toward the top wall, and in the case of bubble collision with the top wall in the opposite direction. The domain of computation is limited to the left-hand side, due to the axisymmetric flow condition.
